# Statins, bone, and neurofibromatosis type 1

**DOI:** 10.1186/1741-7015-6-22

**Published:** 2008-07-31

**Authors:** Bruce R Korf

**Affiliations:** 1 Department of Genetics, University of Alabama at Birmingham, 1530 Third Avenue South, Kaul 230, Birmingham, AL, USA

## Abstract

Neurofibromatosis type 1 (NF1) is a dominantly inherited multi-system disorder. Major features include pigmentary abnormalities, benign tumors of the nerve sheath (neurofibromas), malignant tumors, learning disabilities, and skeletal dysplasia. The *NF1 *gene functions as a tumor suppressor, but haploinsuffiency probably accounts for some aspects of the non-tumor phenotype. The protein product, neurofibromin, is a Ras GTPase-activating protein, and various Ras pathway inhibitors are being tested in preclinical models and clinical trials for effectiveness in treating NF1 complications. This month in *BMC Medicine*, a paper by Kolanczyk et al describes a preclinical mouse model for tibial dysplasia and provides evidence that the drug lovastatin – in use to treat cardiovascular disease – may be beneficial, opening the door to clinical trials in humans.

Patients with neurofibromatosis type 1 (NF1) are largely sustained by the hope that advances in research will result in new forms of treatment. The *NF1 *gene was identified in 1990 and was quickly found to encode a GTPase activating protein (GAP) whose target is the oncoprotein Ras. However, despite great progress in the dissection of cell signaling pathways involved in the disorder, there is still no definitive treatment to prevent or reverse the complications. The path from bench to bedside may be the best approach, but the challenges should not be underestimated. There are, however, recent signs that provide hope that this approach will pay off. The paper by Kolanczyk et al published this month in *BMC Medicine *describes a preclinical mouse model for one of the most difficult NF1 lesions to treat, tibial dysplasia, and provides evidence that an existing drug with a known safety profile in children, lovastatin, may be beneficial, opening the door to clinical trials in humans.

NF1 poses a particular challenge given the complex and highly variable phenotype [1]. The condition is one member of a group of disorders collectively referred to as 'neurofibromatoses', which includes NF1, NF2, and schwannomatosis. Each is associated with a distinct spectrum of nerve sheath tumors, and each is dominantly transmitted, due to mutation in a different gene. The hallmark lesion of NF1 is the neurofibroma, a benign tumor consisting of Schwann cells, fibroblasts, perineurial cells, and mast cells. Patients with NF1 may develop innumerable tumors on the skin, internal tumors, and large disfiguring tumors along major peripheral nerves. Other features include pigmentary lesions (café-au-lait macules, skin-fold freckles), learning disabilities, malignant tumors (gliomas and malignant peripheral nerve sheath tumors), and skeletal dysplasias.

NF1 patients may suffer from both focal and generalized skeletal disorders. Among focal disorders, long bone dysplasia, most commonly involving the tibia, can be a major source of morbidity [2]. Tibial dysplasia presents in infancy with bowing of the lower leg. The dysplastic bone is prone to fracture, and these fractures are very difficult to treat. Pseudoarthrosis ('false joint') may occur, and some patients still require amputation. Non-ossifying fibromas may also occur, sometimes leading to pain or even to fracture. The generalized dysplasia consists of decreased bone mineral density [3-6], as well as slow healing following injury or orthopedic surgery.

Tumors occur in NF1 patients by a classic two-hit model, indicating that the *NF1 *gene is a tumor suppressor [7,8]. A subset of Schwann cells within neurofibromas have mutations of both *NF1 *alleles, the first being the germline mutation and the second being acquired. Large plexiform neurofibromas probably acquire their second hits during development, whereas smaller dermal tumors may acquire theirs later in life. Malignant tumors likely occur upon accumulation of additional genetic changes. At least some non-tumor manifestations also involve a two-hit mechanism: *NF1 *homozygously mutated cells have been found in melanocytes from café-au-lait macules [9] and in cells isolated from dysplastic tibial lesions [10].

Haploinsufficiency of *NF1 *may also play a pathogenetic role for some features. This appears to be the case for the cognitive phenotype, some aspects of which are modeled in mice rendered heterozygous for *Nf1 *mutation (*Nf1 *+/-) [11,12]. It might also explain the diffuse skeletal phenotype of osteopenia. The *NF1 *gene is expressed in chondrocytes, as well as osteoblasts, osteoclasts, and periosteal cells, and the Ras signaling pathway is hyperactive in these cells and in bone marrow-derived osteoprogenitors in *Nf1 *+/- mice [13,14]. The progenitor cells show increased proliferation and decreased ability to differentiate into osteoblasts [15]. The *Nf1 *heterozygous mice do not develop tibial dysplasia, but Kolanczyk et al [16] did obtain mice with tibial bowing by homozygous inactivation of *Nf1 *in developing limbs using a conditional knock-out system. Osteoblasts were found to display increased proliferation but impaired ability to mineralize [16].

Given the role of neurofibromin in stimulating the conversion of Ras-GTP to Ras-GDP, proteins involved in the Ras signal transduction pathway have been identified as potential therapeutic targets. Clinical trials are underway with several existing compounds [17], and several preclinical models have been used in drug testing. Both existing compounds with known action on this pathway, and newly identified compounds are of interest.

The *Nf1 *+/- mouse model has been used, as already noted, to model learning disabilities. The phenotype is rescued by genetic crosses that reduce Ras-GTP in the brain, as well as by treatment with a farnesyl transferase inhibitor, which blocks Ras binding to the cell membrane [18]. Recently, Li et al [19] demonstrated that lovastatin also rescues the cognitive defect, presumably by virtue of its effect on membrane binding of Ras. This has opened the door to clinical trials in children with NF1, since the safety profile of statins in children is known. One negative statin trial has recently been reported [20], and another using a different drug for a longer period of treatment is being planned.

The paper by Kolanczyk et al in this volume of *BMC Medicine *[21] represents the second report of a favorable response to lovastatin in a mouse model, and the first of preclinical treatment aimed at skeletal dysplasia. The conditional knock-out animals display tibial bowing, but do not spontaneously develop fractures, presumably because of different mechanical loads on mouse bones as compared with human. Kolanczyk et al have modeled the tibial fracture by drilling a small hole in the tibia and then monitoring the healing process. Wild-type animals completely heal within 28 days, whereas the knock-out mice display slower and less complete healing. Treatment of the animals with lovastatin resulted in markedly improved bone healing, and normalization of MAPK signaling as compared with untreated animals.

Skeletal dysplasia may thus be another target of lovastatin therapy in clinical trials for patients with NF1. Improvement of bone healing in children with tibial dysplasia would be welcomed, given the enormous difficulty in the management of this complication and the severe associated morbidity. Whether statin treatment will have other benefits related to bone mineral density remains to be determined. Moreover, there is a possibility that these findings will have an impact on the management of bone disorders in non-neurofibromatosis-affected individuals as well. When patient-advocates argue to increase funding for research on neurofibromatosis, one of the potential benefits cited is that this work will shed light on common disorders such as cancer, learning disabilities, and osteoporosis. The NF1 gene clearly plays a role in normal bone development and metabolism, and there is indeed a possibility that pathogenetic and therapeutic insights gained from this rare disorder may someday benefit a much larger population.

## Pre-publication history

The pre-publication history for this paper can be accessed here:


